# Exploring the Benefits of Herbal Medicine Composite 5 (HRMC5) for Skin Health Enhancement

**DOI:** 10.3390/cimb46110720

**Published:** 2024-10-29

**Authors:** Rira Ha, Won Kyong Cho, Euihyun Kim, Sung Joo Jang, Ju-Duck Kim, Chang-Geun Yi, Sang Hyun Moh

**Affiliations:** 1 Department of Beauty Industry, Sungshin Women’s University, Seoul 02844, Republic of Korea; so426611@naver.com (R.H.); jdkim303@sungshin.ac.kr (J.-D.K.); 2 Plant Cell Research Institute of BIO-FD&C Co., Ltd., Incheon 21990, Republic of Korea; wonkyong@gmail.com (W.K.C.); ehkim@biofdnc.com (E.K.); sjjang@biofdnc.com (S.J.J.); 3 College of Medicine, Chung-Ang University, Seoul 06973, Republic of Korea; cgyi0126@gmail.com

**Keywords:** traditional Korean herbal extracts, anti-inflammatory properties, skin barrier function, wound healing, UV protection

## Abstract

The skin, as the body’s largest organ, is vital for protecting against environmental stressors, regulating temperature, and preventing water loss. Here, we examined the potential of a mixture of five traditional Korean herbal extracts—*Cimicifuga racemosa*, *Paeonia lactiflora*, *Phellodendron amurense*, *Rheum rhaponticum*, and *Scutellaria baicalensis*—referred to as herbal medicine composite 5 (HRMC5) for enhancing skin health and managing menopausal symptoms. High-performance liquid chromatography identified 14 bioactive compounds, including flavonoids, phenolic acids, anthraquinones, and alkaloids. In vitro studies revealed an optimal concentration of 0.625 g/L for cell survival and UV protection, with the mixture demonstrating significant wound-healing properties comparable to epidermal growth factor. HRMC5 exhibited anti-inflammatory effects by downregulating *COX2* expression and upregulating the key skin barrier proteins. A 4-week clinical trial involving 20 postmenopausal women showed significant improvements in skin redness, hemoglobin concentration, and skin moisture content. Visual analog scale assessments indicated substantial reductions in facial flushing severity and the associated sweating. The topical application of HRMC5 cream offered potential advantages over ingested phytoestrogens by reducing the systemic side effects. These findings suggest that HRMC5 is a promising non-invasive treatment for vasomotor symptoms in menopausal women and overall skin health, warranting further research on its long-term efficacy and safety in larger populations.

## 1. Introduction

The skin, as the body’s largest organ, plays a crucial role in protecting against environmental stressors, regulating temperature, and preventing water loss [1]. The skin barrier is composed of lipids and proteins that make up the stratum corneum, which functions to maintain skin hydration and protect against external stimuli [2]. Maintaining a healthy skin barrier is essential for the overall skin health and function [3]. Facial flushing, characterized by sudden reddening of the face, neck, and upper chest, is a common dermatological phenomenon caused by increased blood flow to the skin’s surface [4]. This condition can be triggered by various factors, including emotional stress, hormonal changes, alcohol consumption, spicy foods, certain medications, and genetic factors [5,6,7,8]. While often benign, facial flushing can also be a symptom of underlying medical conditions such as rosacea, carcinoid syndrome, or thyroid disorders [9]. Diagnosis typically involves a thorough medical history, physical examination, and the consideration of the associated symptoms, with additional tests performed if necessary [10,11]. Management strategies vary depending on the cause, ranging from simple lifestyle modifications and topical treatments to medications addressing the specific underlying conditions [12]. Although usually harmless, persistent or severe flushing should be evaluated by a healthcare professional to rule out more serious issues. Understanding the diverse causes and appropriate management of facial flushing is crucial for both patients and healthcare providers in addressing this common yet potentially distressing condition [13].

Menopause is a critical transitional period in a woman’s life, marked by significant hormonal changes and the cessation of reproductive function [14]. This phase, which typically occurs between the ages of 45 and 55, affects millions of women worldwide and is characterized by a variety of physical and psychological symptoms [15]. As estrogen levels decline, women may experience hot flushes, night sweats, mood swings, sleep disturbances, and changes in the skin quality [16]. The management of menopausal symptoms is crucial for maintaining the quality of life and overall health during this period [17]. While hormone replacement therapy has been a common treatment, there is growing interest in natural alternatives, including herbal remedies and dietary supplements, to alleviate menopausal symptoms and support skin health [18].

Herbal medicine encompasses the use of plant-based remedies for disease prevention and treatment, spanning from traditional practices in various cultures to modern, standardized herbal extracts [19]. Herbal medicines are often region-specific, as they rely on plants native to or commonly grown in particular geographical areas. While India and China are widely recognized as the two main powerhouses of traditional herbal medicine, with their well-established systems of Ayurveda and traditional Chinese medicine, respectively, many countries around the world have developed their own unique traditional medicinal practices based on the local flora [20].

Traditional Korean herbal medicine, known as Hanbang in Korean, has been an integral part of Korean culture for centuries, utilizing herbs to treat various ailments and promote overall health both domestically and internationally [21]. In recent years, there has been growing interest in exploring the potential of these traditional remedies for modern applications, particularly in the field of dermatology [22]. The research on Korean cosmeceuticals emphasizes the innovative use of natural ingredients with antiaging, antitumor, and antimelanogenic effects, while also analyzing the composition and metal content of Korean herbs and herbal products to ensure their safety and efficacy in modern dermatological applications [23,24]. Moreover, instead of using a single extract, a mixture of Korean herbal extracts could be effectively applied for the treatment of inflammatory skin diseases [25].

The skin health of individuals experiencing hormonal changes has become a significant concern in recent years, with issues such as facial flushing and skin barrier damage leading to both physical and psychological distress [26]. Despite the growing need, there has been a lack of comprehensive research addressing these specific skin problems in this demographic. Concurrently, the consumer demand for natural ingredient-based cosmetics has surged, driven by the expanding clean beauty and vegan beauty trends.

As a result, it is important to develop and evaluate the efficacy of a natural-derived cosmetic product specifically designed for skin issues related to facial flushing and skin barrier damage. For that, we developed herbal medicine composite 5 (HRMC5), a complex extract comprising five traditional Korean herbal extracts. These extracts include *Cimicifuga racemosa* root extract, *Paeonia lactiflora* root extract, *Phellodendron amurense* root extract, *Rheum rhaponticum* root extract, and *Scutellaria baicalensis* bark extract. These ingredients were selected based on their specific properties that contribute to skin health as follows. *Cimicifuga racemosa* offers anti-inflammatory and vasoconstriction effects, potentially alleviating skin redness [27]. *Paeonia lactiflora* provides vasoconstrictive and anti-inflammatory actions [28]. *Phellodendron amurense* offers anti-inflammatory and antimicrobial effects, potentially helping to calm the skin and reduce irritation [29]. *Rheum rhaponticum* is known for its antioxidant and anti-inflammatory properties [30]. *Scutellaria baicalensis* possesses strong anti-inflammatory and antioxidant effects, which may help calm the skin and reduce sensitivity [31].

We chose to combine these five extracts to explore the potential synergistic effects that could enhance skin health beyond what individual extracts achieve. Our comprehensive approach demonstrated that this combination offers superior anti-inflammatory and barrier-strengthening properties compared to single extracts at equivalent concentrations. This is consistent with previous research showing that combinations of active ingredients can provide enhanced benefits for skin health. For example, studies have shown that combining vitamin C with other antioxidants can improve its efficacy in protecting against UV damage and promoting collagen production [32]. Additionally, our approach aligns with the growing body of evidence supporting the use of multi-ingredient formulations to address the various aspects of skin health, including hydration, elasticity, and reduction in hyperpigmentation [33]. By leveraging the unique properties of each extract in HRMC5, we aimed to create a comprehensive solution for improving the overall skin health and managing menopausal symptoms, particularly facial flushing.

We employed a comprehensive approach to evaluate HRMC5’s efficacy, utilizing high-performance liquid chromatography (HPLC) to identify its bioactive compounds, and conducting in vitro studies to assess the cell viability, UV protection, and wound healing properties. Gene expression analyses revealed HRMC5’s anti-inflammatory effects and its ability to upregulate the key skin barrier proteins. A 4-week clinical trial with 20 postmenopausal women demonstrated HRMC5’s effectiveness in managing menopausal symptoms, particularly in alleviating facial flushing. Our study showed significant improvements in skin redness, hemoglobin concentration, and skin moisture content. Visual analog scale assessments confirmed substantial reductions in facial flushing severity and the associated sweating. These findings collectively indicate that HRMC5 is effective in reducing skin redness, enhancing the skin barrier, and managing the vasomotor symptoms in menopausal women.

## 2. Materials and Methods

### 2.1. Preparation of Five Herbal Plant Extracts

Five herbal materials were used: *Cimicifuga racemosa* root extract, *Paeonia lactiflora* root extract, *Phellodendron amurense* root extract, *Rheum rhaponticum* root extract, and *Scutellaria baicalensis* bark extract. Fifty grams of each plant material were extracted using 1 L of 70% ethanol at 60 °C for 24 h. We selected 70% ethanol as the extraction solvent based on preliminary experiments showing the optimal extraction of both polar and non-polar compounds. The extraction temperature of 60 °C was chosen to balance the extraction efficiency with compound stability, particularly for heat-sensitive bioactive molecules. The extracted solution was filtered, concentrated using a rotary vacuum evaporator, and finally freeze-dried into a powder form. Each plant extract was mixed in equal proportions and used for further analysis. This composite sample was prepared to represent the combined effects of all the five extracts. To evaluate the effectiveness and safety of the extracts, the solution was diluted to various concentrations. The initial concentration of 5 g per liter was deemed high and potentially toxic, so it was diluted to 2.5 g per liter, 1.25 g per liter, 0.625 g per liter, 0.313 g per liter, 0.156 g per liter, and 0.078 g per liter. For the cell culture experiments, the extracts were further diluted to ensure that the ethanol concentration did not exceed 1%, to avoid the potential toxicity to the cells.

### 2.2. Sample Preperation for HPLC

The mixed herbal plant extract was re-dissolved in 70% ethanol at a concentration of 5 g/L. This concentration was necessary to adjust the optimal detection range for HPLC analysis. The re-dissolved sample was filtered through a 0.45 μm polytetrafluoroethylene (PTFE) syringe filter to eliminate any remaining particulates that might interfere with the HPLC analysis.

### 2.3. HPLC Analysis

The HPLC analysis was performed using an Arc HPLC system, which was equipped with a 2998 Photodiode Array (PDA) detector from Waters, USA. The separation of the components was achieved using a Shim-Pack GIS C18 column (4.6 × 250 mm, 5 μm, Shimadzu, Japan). The mobile phases used were: Mobile Phase A, which consisted of water containing 0.1% trifluoroacetic acid (TFA), and Mobile Phase B, which consisted of acetonitrile containing 0.1% TFA.

For the operational parameters, a 20 μL volume of each prepared sample was injected. The column was maintained at a temperature of 30 °C. The detector settings were configured to scan from 200 to 800 nm.

The gradient program was as follows: at 0 min, the mobile phase composition was 90% A and 10% B. Until 20 min, this composition was maintained at 90% A and 10% B. By 40 min, the composition changed to 80% A and 20% B. By 60 min, it shifted to 60% A and 40% B. By 80 min, the composition shifted to 30% A and 70% B. By 82 min, the composition changed to 10% A and 90% B, which was maintained until 95 min. From 95 to 97 min, the composition returned to 90% A and 10% B. Finally, until 110 min, the composition remained at 90% A and 10% B.

### 2.4. Data Acquisition and Analysis

Peak identification was performed by comparing the chromatographic peak retention times and spectral data with those of the individual plant extracts. Each peak was then identified and confirmed using BIO-FD&C Co., Ltd.’s proprietary natural product database (Incheon, South Korea). 

### 2.5. Assessment of the Cellular Viability

The impact of the herbal extracts on the cell proliferation and survival was evaluated using the Cell Counting Kit-8 (CCK-8) assay. Human keratinocytes (HaCaT) were plated in 96-well formats at 5 × 10^4^ cells per well and cultured for 24 h. Subsequently, the cells were exposed to varying concentrations of herbal extracts (5 g per liter, 2.5 g per liter, 1.25 g per liter, 0.625 g per liter, 0.313 g per liter, 0.156 g per liter, and 0.078 g per liter) for an additional 24 h, with sterile water serving as a control. Following treatment, 1× CCK-8 solution (Catalog Number CCK-3000, Donginbio, Seoul, Republic of Korea) was introduced to each well, and the cells were incubated for 3 more hours. Absorbance readings at 450 nm were obtained using a Thermo Scientific Multiskan GO Microplate Spectrophotometer (Fisher Scientific Ltd., Vantaa, Finland). The cell viability was quantified as a percentage, calculated by dividing the absorbance of the treated cells by that of the control cells and multiplying by 100.

### 2.6. Evaluation of the Ultraviolet Protection

Human adult keratinocyte (HaCaT) cells were seeded in 96-well plates at 5 × 10^4^ cells per well and grown for 24 h. The culture medium was then replaced with serum-free medium for 4 h. The cells were subjected to UVB radiation (5–15 mJ/cm^2^) using a UVP CL-1000 Ultraviolet Crosslinker. Post-irradiation, the cells were treated with herbal extracts and incubated for 24 h. A CCK-8 assay was subsequently performed to assess the alterations in the cell viability.

### 2.7. Analysis of the Wound Healing Capacity

The wound healing process was evaluated using specialized culture inserts placed in 24-well plates. HaCaT cells were introduced into the inserts at a density of 3 × 10^5^ cells per well and cultured until they reached approximately 90% confluence, which took 24 h. Once the inserts were removed, the initial cell arrangement was photographed to establish a baseline. The cells were subsequently exposed to 100 ng/mL of epidermal growth factor (EGF), applied to both the experimental samples and positive control groups. After an 18 h incubation period, the cells underwent fixation using 4% paraformaldehyde for 15 min. They were then rinsed with phosphate-buffered saline (PBS) before being imaged a second time. The progression of wound healing was quantified by measuring the cell migration between the initial (0 h) and final (18 h) images using ImageJ software (version 1.54j).

### 2.8. Transcriptional Analysis via Quantitative RT-PCR

The cells exposed to herbal extracts were harvested using trypsin, rinsed with PBS, and cryopreserved at −80 °C pending RNA isolation. A minimum of 1 × 10^6^ cells per experimental group underwent RNA extraction using the RNeasy Mini kit (Qiagen, Hilden, Germany). mRNA concentrations were quantified using a NanoDrop spectrophotometer. cDNA synthesis was performed using RT Master Mix 10 (Qiagen). Quantitative PCR was executed using THUNDERBIRD Next SYBR qPCR Mix (Toyobo, Osaka, Japan), specific primers, and the synthesized cDNA. The thermal cycling protocol consisted of 40 cycles: denaturation (95 °C, 15 s), annealing (62 °C, 60 s), and extension (72 °C, 60 s). The gene expression was normalized to glyceraldehyde-3-phosphate dehydrogenase (GAPDH), and the relative expression levels were computed using the R = 2^−[ΔΔCt]^ method.

### 2.9. Protein Localization Analysis

Following the herbal extract treatment, the cells underwent PBS washing and fixation with 4% paraformaldehyde for 60 min. Subsequently, the cells were permeabilized using 1% Triton X-100 and blocked with 2% bovine serum albumin to prevent non-specific antibody binding. The primary antibodies targeting Involucrin (1:200), Claudin1 (1:200), Filaggrin (1:100), and Collagen Type I (1:200) were applied and incubated overnight at 4 °C. After washing, the cells were treated with a fluorescein isothiocyanate-labeled secondary antibody and counterstained with Hoechst-33342. Fluorescence imaging was performed using a fluorescence microscope, and the resulting images were analyzed using ImageJ software (version 1.54j).

### 2.10. Preparation of the HRMC5 Cream Formulation

The HRMC5 cream formulation was prepared through a multi-step process (A, B, C1, C2, D, E, and F). Initially, phase A, consisting of water, carbomer, and disodium EDTA, was completely dissolved. This was then combined with phase B, which contained additional water, glycerin, butylene glycol, and sodium hyaluronate solution, and heated to 75 °C in the main tank. Concurrently, phase C1, a complex mixture of various oils and emulsifiers including sorbitan stearate, glyceryl stearate, cetearyl alcohol, and several plant-derived oils, was heated separately to 75 °C until fully dissolved. The aqueous phase (A + B) and oil phase (C1, C2) were then emulsified using a homo mixer at 3000 rpm for 3 min, with phase C2, composed solely of cyclopentasiloxane, added just before the emulsification. After the emulsification, the mixture was cooled to 50 °C, and phase D, a solution of water and tromethamine, was added. The mixture was neutralized by mixing at 3000 rpm for 3 min. Subsequently, phase E (1,2-hexanediol and ethylhexylglycerin) was introduced and mixed at 2500 rpm for 2 min, followed by the addition of phase F (HRMC5) and mixing at the same speed for an additional 2 min. The complete mixture was then cooled to 25 °C, at which point its hardness, pH, and specific gravity were measured to confirm its physical properties.

### 2.11. Human Application of the HMRC5 Cream Formulation

Our study involved participants using the HRMC5 cream for four weeks. Prior to starting, the participants completed a menopausal symptom self-assessment questionnaire. Measurements of skin redness (a*), hemoglobin concentration and uniformity, skin moisture content, and skin moisture images were taken at the baseline, after two weeks, and after four weeks of product use. A Visual Analog Scale (VAS) was employed to assess the reduction in facial flushing. Our study also included surveys on product efficacy, usability, and skin safety. This research was conducted under IRB approval number CDIRB-RR-23-002.

Our study recruited women aged 50–59 experiencing menopausal symptoms. Strict eligibility criteria were applied, excluding those who were pregnant, breastfeeding, or had various health conditions that could interfere with the study. The participants were required to be free from recent use of specific topical treatments, cosmetic products, or dermatological procedures in the test area. From an initial selection of 22 participants, 20 completed the study, with an average age of 53.6 ± 3.1 years.

The participants assessed their menopausal status using a self-evaluation based on the Kupperman index (KI) before using the product [34]. The evaluation included 11 items totaling 0–51 points: 5–9 points indicated mild menopause, 10–14 points moderate menopause, and 15 points or more severe menopause.

### 2.12. Measurement of the Skin Redness (a*)

The skin redness (a*) was measured using the VISIA-CR2.2 facial imaging device from Canfield (Parsippany, NJ, USA). The entire face was captured in the optical mode, and the images were analyzed using the Image-pro analysis program from MediaCybernetics (Rockville, MD, USA) to assess the skin redness in the cheek area. A decrease in the skin redness (a*) indicates an improvement in facial flushing. In this study, the left and right cheek areas were analyzed before product use, after 2 weeks of use, and after 4 weeks of use.

### 2.13. Measurement of the Hemoglobin Concentration and Uniformity

The hemoglobin concentration and uniformity were measured using the Antera 3D CS device from Miravex Limited (Dublin, Ireland). This device enhances the pore visualization and quantifies the related characteristics such as density and granularity by capturing high-resolution images of the skin surface and converting them into 3D images. In this study, the left and right cheek areas were photographed before product use, after 2 weeks of use, and after 4 weeks of use, with analyses conducted in the hemoglobin mode. A decrease in parameter values indicates an improvement in facial flushing.

### 2.14. Measurement of the Skin Moisture Content (Hydration)

The skin moisture content (hydration) was measured using the Corneometer CM825 from Courage + Khazaka electronic GmbH, (Köln, Germany), which measures the water content in the stratum corneum based on the capacitance principles. Higher measured values indicate a higher moisture content. In this study, the left or right cheek area was measured three times before product use, after 2 weeks of use, and after 4 weeks of use, with the average values analyzed. Skin moisture images were also captured using the MoistureMap MM100 from Courage + Khazaka electronic GmbH.

### 2.15. Vas Evaluation for the Facial Flushing Relief

The participants evaluated the degree of hot flushes and sweating due to menopause using a 10-point VAS during each visit. The participants marked their perceived level of facial flushing and sweating on a 10 cm line segment, with measurements taken before product use, after 2 weeks of use, and after 4 weeks of use. The scale ranged from 0 (no facial flushing/sweating) to 10 (severe and unbearable facial flushing/sweating).

### 2.16. Participant Survey Evaluation and Skin Safety Evaluation

After the product’s use, the participants completed a self-assessment survey regarding the product’s efficacy and usability. The evaluation used a 6-point scale ranging from “Strongly disagree” to “Strongly agree”, with the positive responses analyzed. Each participant’s test area was observed and its condition was recorded through interviews. Any adverse reactions to the product were documented in an adverse reaction report, with the principal investigator determining their relationship to the test product.

### 2.17. Statistical Analysis and Data Interpretation

The statistical evaluation of the data was performed using SigmaStat software version 4.0 (SPSS, Inc., Chicago, IL, USA). Each experiment was replicated at least three times, incorporating both biological and technical replicates to ensure robustness. Results are presented as mean ± standard error. Prior to analysis, all data sets were examined for normality and homogeneity of variance. For comparisons involving three or more groups, Kruskal-Wallis tests were applied to non-parametric data, while one-way analysis of variance (ANOVA) was used for parametric data. Post-hoc analyses following ANOVA included Duncan’s multiple range test (for equal variances) or Dunnett’s T3 test (for unequal variances). For comparisons between two groups, Mann-Whitney U tests (for non-parametric data) or Student’s *t*-tests (for parametric data) were utilized. The significance threshold was set at *p* < 0.05 for all analyses. Visual representations of the data, including graphs and charts, were generated using GraphPad PRISM 5.01 (GraphPad Software, USA). This rigorous statistical approach was applied consistently across all experiments, including the evaluation of HRMC5 on multiple skin parameters and menopausal symptoms, ensuring a comprehensive and reliable analysis of the study results.

## 3. Results

### 3.1. HPLC Analysis and Identification of the Bioactive Compounds in a Mixture of Five Traditional Korean Herbal Extracts

We examined the chemical compounds of five traditional Korean herbal extracts (HRMC5) using the HPLC analysis. HRMC5 was created by combining equal proportions of extracts from *Cimicifuga racemosa*, *Paeonia lactiflora*, *Phellodendron amurense*, *Scutellaria baicalensis*, and *Rheum rhaponticum*, each individually extracted using 70% ethanol.

**Figure 1:**
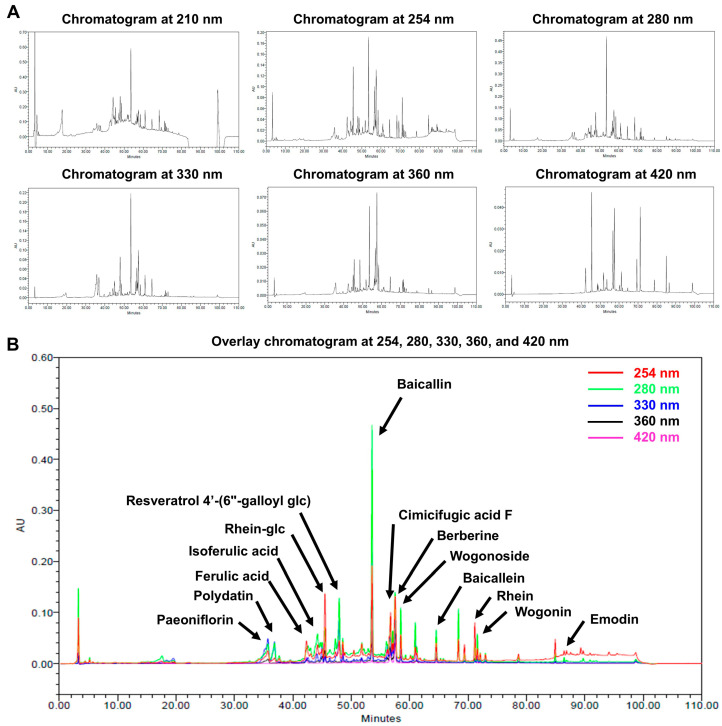
HPLC analysis of a mixture of five traditional Korean herbal medicine extracts, collectively referred to as HRMC5. (**A**) Chromatograms of the extract at different wavelengths (210, 254, 280, 330, 360, and 420 nm). (**B**) Overlay chromatogram of the extract, showing peaks corresponding to 14 identified compounds.

We observed the chromatograms at six different wavelengths ranging from 210 nm to 420 nm (Figure 1A). Peaks also appeared in the visible region at 420 nm, likely contributing to the overall yellowish color of the extract. Excluding the results from 210 nm, the intensity was the highest at 280 nm, followed by 254 nm and 330 nm. The chromatogram observed at 254 nm showed various peaks of appropriate size, suggesting that this wavelength would be the most suitable for the analysis.

The HPLC analysis successfully identified fourteen distinct bioactive compounds collectively referred to as HRMC5 (Figure 1B and Table 1). The peak intensity was the highest at 210 nm; however, due to the baseline noise caused by the low wavelength, this wavelength was excluded when constructing the overlay chromatogram (Figure 1B). Even excluding 210 nm, a total of fourteen compounds were identified at various wavelengths of 254 nm, 280 nm, 330 nm, 360 nm, and 420 nm. The analysis revealed that the mixture contained polar compounds, such as paeoniflorin, polydatin, isoferulic acid, and ferulic acid, which were eluted early in the chromatographic separation (Table 1). Prominent flavonoids from *Scutellaria baicalensis*, including baicalin, baicalein, and wogonoside, were identified in the middle retention times. Anthraquinones rhein and emodin from *Rheum rhaponticum* and the alkaloid, berberine, from *Phellodendron amurense* were detected at the later retention times.

We also examined the known properties of the identified compounds (Table 1). Baicalin, baicalein, wogonin, and wogonoside are flavonoids known for their antioxidant and anti-inflammatory properties. Ferulic acid and isoferulic acid are phenolic acids with similar properties. Cimicifugic acid F, found in the *Cimicifuga* genus, is known for its potential therapeutic effects on menopausal symptoms. Paeoniflorin is a monoterpene glycoside with potential anti-inflammatory and analgesic properties. Emodin and rhein are anthraquinone derivatives with potential anti-inflammatory and laxative effects. Resveratrol 4′-(6″-galloyl glc) is a stilbene compound known for its antioxidant and antiaging properties. Rhein-glc and polydatin are glycosides of rhein and resveratrol, respectively.

### 3.2. Effect of HRMC5 on Cell Survival and UV Protection

**Figure 2:**
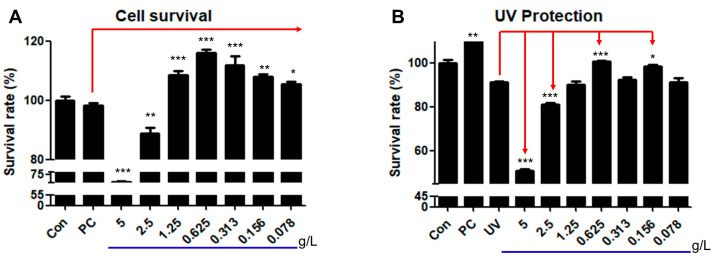
Effects of HRMC5 on cell survival and UV protection. (**A**) Survival rate of cells treated with different concentrations of herbal medicine extract. (**B**) Survival rate of cells treated with herbal medicine extract after UV exposure. Sterile water was utilized as the control while absolute ethanol 1% was used as the positive control (PC). The statistical significance indicators (*, **, and ***) are based on the specified levels (0.05, 0.01, and 0.001), respectively.

We examined the effect of HRMC5 on cell survival and UV protection (Figure 2). The mixture showed a biphasic effect on cell survival (Figure 2A). For example, at low concentrations (0.078–1.25 g/L), the extract significantly increased the cell survival compared to the control group. At higher concentrations (2.5–5 g/L), the extract exhibited cytotoxic effects, reducing the cell survival significantly. The best results (the highest cell survival) were seen at a concentration of 0.625 g/L.

Regarding UV protection, HRMC5 did not show any recovery from UV-induced damage at concentrations of 5, 2.5, or 1.25 g/L (Figure 2B). However, consistent with the cell viability results, the extract at a concentration of 0.625 g/L significantly protected the cells from UV damage. Given these findings, we decided to continue our future experiments using a concentration of 0.625 g/L of the mixture.

### 3.3. Effect of HRMC5 on Wound Healing

**Figure 3:**
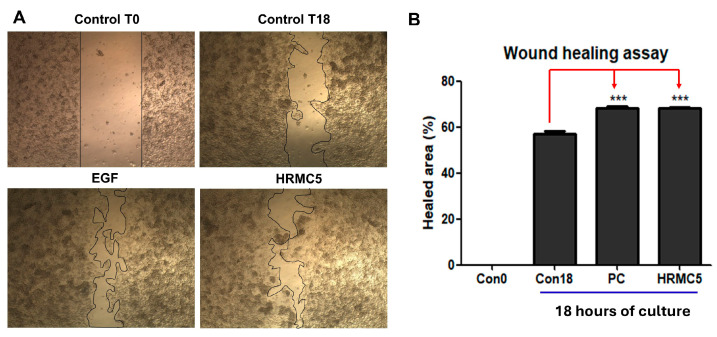
Effect of HRMC5 on wound healing. (**A**) Microscopic images showing the wound-healing process at different time points and under different treatment conditions. Control T0: untreated control at 0 h after scratch. Control T18: untreated control at 18 h after scratch. EGF: positive control treated with epidermal growth factor (100 ng/mL). HRMC5: experimental group treated with a mixture of five herbal medicine extract compounds (0.625 g/L). (**B**) The percentage of healed area after 18 h of culture for different treatment groups. Statistical significance was determined using ANOVA followed by Tukey’s post-hoc test. *** *p* < 0.001 compared to control.

The wound healing assay demonstrated the significant effect of HRMC5, a mixture of five herbal medicine extract compounds, on promoting wound closure. Microscopic images revealed that after 18 h, the untreated control group showed only partial healing, whereas the group treated with epidermal growth factor (EGF), a known positive control, exhibited nearly complete wound closure (Figure 3A). Similarly, the HRMC5-treated group also showed substantial healing, comparable to the EGF-treated group (Figure 3A). Quantitative analysis confirmed that both the EGF and HRMC5 groups achieved approximately 68.4% wound healing after 18 h, while the untreated control displayed only around 57.1% healing. Statistical analysis using ANOVA followed by Tukey’s post-hoc test indicated that the improvements observed in the HRMC5 and EGF groups were highly significant compared to the control (*** *p* < 0.001). These results highlight the potent wound-healing properties of HRMC5, suggesting that it may be as effective as EGF in enhancing wound repair.

### 3.4. Effect of HRMC5 on the Skin Barrier and Inflammation Genes

**Figure 4:**
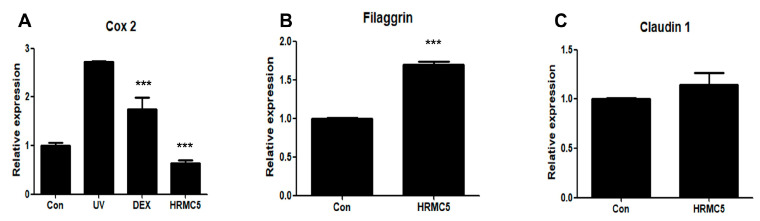
Effect of HRMC5 on *COX2*, *Filaggrin*, and *Claudin 1* gene expression. (**A**) The relative expression of *COX2*, an inflammatory marker, in various treatment groups. The control group received no treatment, while the UV group was exposed to ultraviolet radiation only. The positive control group was treated with dexamethasone (DEX), a known anti-inflammatory agent. The experimental group was treated with HRMC5. (**B**) The relative expression of *Filaggrin* encoding a skin barrier protein, in different treatment groups. The control group received no treatment, while the experimental group was treated with HRMC5. (**C**) The relative expression of *Claudin 1* encoding another skin barrier protein, in various treatment groups. The control group received no treatment, while the experimental group was treated with HRMC5. Statistical significance was determined using ANOVA followed by Tukey’s post-hoc test. *** *p* < 0.001 compared to control.

HRMC5 demonstrated a significant anti-inflammatory effect, as indicated by the statistically significant downregulation of *COX2* expression in the HRMC5-treated group compared to the UV-exposed group (*** *p* < 0.001) (Figure 4A). This suggests that HRMC5 effectively reduces the inflammatory responses in the skin. While HRMC5 showed a trend towards increasing the expression of *Filaggrin*, a key gene involved in skin barrier function, this effect was also statistically significant (*** *p* < 0.001) (Figure 4B). This could imply that HRMC5 might have a weaker or more indirect influence on the skin barrier formation compared to its anti-inflammatory properties. There were no significant changes in *Claudin 1* expression, another skin barrier gene, between the HRMC5-treated group and the control group (Figure 4C). This suggests that HRMC5 primarily affects the skin barrier function through its modulation of *Filaggrin*, rather than *Claudin 1*. Overall, HRMC5 exhibited a potent anti-inflammatory effect, as evidenced by the statistically significant downregulation of *COX2* expression (*** *p* < 0.001).

### 3.5. Effect of HRMC5 on the Skin Barrier Proteins

**Figure 5:**
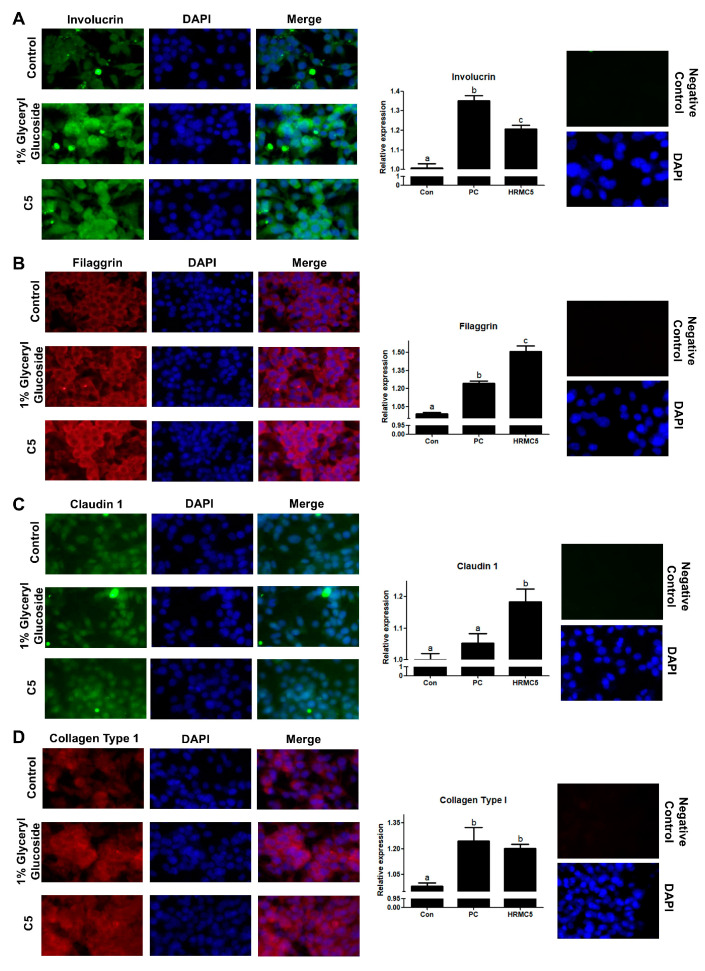
Effect of HRMC5 on skin barrier proteins. (**A**–**D**) Left panels: representative immunofluorescence staining images of (**A**) Involucrin, (**B**) Filaggrin, (**C**) Claudin 1 (all green), and (**D**) Collagen Type 1 (red) in keratinocytes treated with different concentrations of C5 or positive control (1% Glyceryl glucoside). DAPI (blue) stains cell nuclei. Right panels: quantification of protein expression relative to the control group for (**A**) Involucrin, (**B**) Filaggrin, (**C**) Claudin 1, and (**D**) Collagen Type 1. Statistical significance was determined using ANOVA followed by Tukey’s post-hoc test. Different letters indicated significant difference between groups (*p* < 0.05).

We examined the effects of 1% glyceryl glucoside and HRMC5 on the expression of several skin barrier proteins, including Involucrin, Filaggrin, Claudin 1, and Collagen Type 1 (Figure 5). Immunofluorescence analysis shows that both treatments significantly upregulated Involucrin and Filaggrin expression compared to the control, with HRMC5 producing the highest levels for both proteins (Figure 5A,B). Claudin 1 expression also increased significantly in the HRMC5-treated group compared to both the control and 1% glyceryl glucoside, highlighting HRMC5’s strong effect (Figure 5C). Similarly, Collagen Type 1 levels were significantly elevated in both treated groups compared to the control, though there was no significant difference between the effects of HRMC5 and 1% glyceryl glucoside on this protein (Figure 5D). Overall, HRMC5 showed a greater enhancement of the skin barrier proteins, particularly Involucrin, Filaggrin, and Claudin 1, compared to the 1% glyceryl glucoside treatment. 

### 3.6. Study Design and Participant Characteristics in the HRMC5 Cream Evaluation for Menopausal Facial Flushing

We evaluated the effects of a cream containing HRMC5 on the facial flushing in menopausal women over a 4-week period, measuring the skin redness, hemoglobin concentration, hemoglobin unevenness, and skin moisture content. All 20 participants were in their 50s, with 10% in moderate menopause and 90% in severe menopause (Table 2). Regarding the skin characteristics, 55% had dry skin, 35% normal, and 10% a combination. The facial moisture was normal for 65%, lacking for 30%, and very lacking for 5% of the participants. The facial oil levels were normal for 80% and lacking for 20%, while the body dryness was reported as normal by 55% and dry by 45%. Most of the participants (60%) showered once daily, 75% occasionally used body products, and 75% had 1–3 h of daily UV exposure. The majority (90%) slept 5–8 h per day. Notably, none of the participants reported skin irritation, sensitivity, or adverse reactions to skincare products. Regarding the menstrual-related skin changes, 55% reported no changes, while 45% were not applicable, likely due to being post-menopausal. These comprehensive results provide a detailed profile of the study participants, offering insights into their menopausal status, skin characteristics, and lifestyle factors that could influence the effectiveness of the HRMC5-containing cream in alleviating facial flushing.

### 3.7. Efficacy of the HRMC5-Containing Cream on Menopausal Symptoms and Skin Parameters

**Figure 6:**
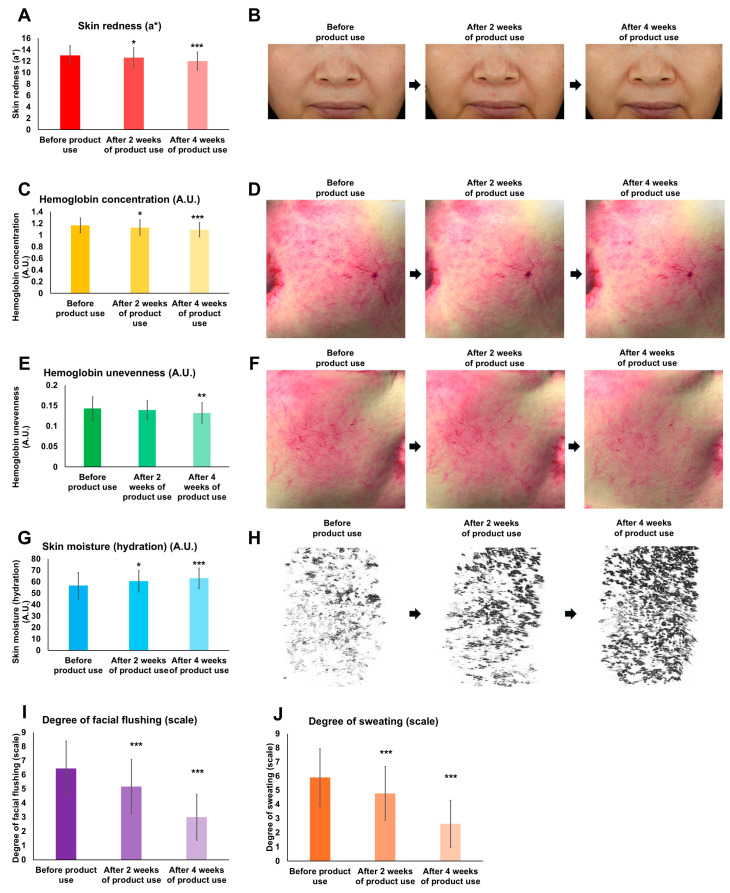
Effects of HRMC5-containing cream on various skin parameters over 4 weeks of use. HRMC5-containing cream was applied to participants, and individual skin parameters were examined at baseline, 2 weeks, and 4 weeks after initiating product use. (**A**) Quantitative analysis of skin redness reduction over time. (**B**) Representative images demonstrating visible reduction in skin redness at baseline, 2 weeks, and 4 weeks. (**C**) Graph showing the decrease in hemoglobin concentration in the skin over the study period. (**D**) Representative images illustrating the reduction in hemoglobin concentration at baseline, 2 weeks, and 4 weeks. (**E**) Quantitative assessment of hemoglobin distribution evenness improvement. (**F**) Representative images showing the progression of hemoglobin distribution evenness at baseline, 2 weeks, and 4 weeks. (**G**) Graph depicting the increase in skin moisture content over time. (**H**) Representative images illustrating improved skin hydration at baseline, 2 weeks, and 4 weeks. (**I**) Quantitative analysis of facial flushing severity reduction throughout the study period. (**J**) Graph showing the decrease in sweating intensity over the course of the study. All data are presented as mean ± standard deviation (n = 20). * *p* < 0.05, ** *p* < 0.01, and *** *p* < 0.001 compared to the baseline measurements (before product use).

We demonstrated significant improvements across multiple skin parameters and menopausal symptoms following the use of the HRMC5-containing cream. The skin redness on the cheeks decreased progressively, showing a 3.00% reduction after 2 weeks (* *p* < 0.05) and a 7.52% reduction after 4 weeks of product use (*** *p* < 0.001) (Figure 6A,B). Similarly, the hemoglobin concentration in the cheeks reduced by 3.36% and 6.56% after 2 and 4 weeks, respectively, with a significant improvement observed at 4 weeks (*** *p* < 0.001) (Figure 6C,D). Uneven hemoglobin distribution decreased by 6.94% after 4 weeks (** *p* < 0.01), indicating an improved hemoglobin uniformity (Figure 6E,F). The skin moisture levels increased significantly, with an 8.65% rise after 2 weeks and a 13.45% rise after 4 weeks (*** *p* < 0.001) (Figure 6G,H). Notably, the VAS evaluation revealed substantial reductions in menopausal facial flushing (20.88% after 2 weeks, 52.96% after 4 weeks) and the associated sweating (20.45% after 2 weeks, 54.75% after 4 weeks) (*** *p* < 0.001 for all) (Figure 6I,J). Our results collectively demonstrate the cream’s effectiveness in improving the facial redness and skin hydration, and alleviating menopausal symptoms such as facial flushing and sweating.

## 4. Discussion

The HPLC analysis successfully identified 14 bioactive compounds in HRMC5. These compounds, including flavonoids, phenolic acids, anthraquinones, and alkaloids, each contribute to the therapeutic potential of the mixture. Flavonoids such as baicalin, baicalein, and wogonoside from *Scutellaria baicalensis* are known for their strong antioxidant and anti-inflammatory properties [35]. Phenolic acids like ferulic and isoferulic acid further enhance the antioxidant capacity of the mixture [36]. The presence of anthraquinones such as rhein and emodin from *Rheum rhaponticum* contributes antimicrobial and anti-inflammatory effects [37], while berberine from *Phellodendron amurense* offers antibacterial benefits [38,39]. The study emphasizes the potential synergistic effects of these bioactive compounds, suggesting an enhanced therapeutic efficacy when used in combination.

The HPLC analysis was conducted at multiple wavelengths, with 254 nm identified as the most suitable for the comprehensive detection of phytochemicals. Our study highlights the potential synergistic effects of the identified compounds, suggesting an enhanced therapeutic efficacy when combined. The findings align with the previous research on individual herbs, reinforcing the bioactive profiles of these traditional remedies [40,41]. Future studies employing advanced analytical techniques could further elucidate the full phytochemical profile and pharmacological activities of this herbal mixture [42].

The mixture demonstrated concentration-dependent effects on the cell viability and UV protection, with a biphasic response observed. Lower concentrations (0.078–1.25 g/L) improved the cell survival, while higher concentrations (2.5–5 g/L) exhibited cytotoxic effects. Such biphasic effects of plant extracts have been demonstrated in other previous studies [43,44]. The optimal concentration for both the cell survival and UV protection was found to be 0.625 g/L. This finding highlights the mixture’s narrow therapeutic window, where the beneficial effects are only observed at specific concentrations as shown previously [45]. The extract’s ability to protect the cells from UV-induced damage further indicates its potential as a cytoprotective agent, particularly at the optimal concentration.

The microscopic images clearly demonstrated that the mixture significantly enhanced the wound-healing process compared to the untreated control groups [46]. Notably, the wound-healing properties of the herbal mixture were significant, as evidenced by the enhanced wound closure comparable to the effects of EGF [47]. The quantitative analysis of the healed area after 18 h of culture further confirmed these observations, with the mixture showing a statistically significant improvement in wound closure compared to the untreated controls. This result supports the potential of the mixture as a natural alternative or complementary treatment for promoting wound healing. The observed wound-healing efficacy suggests that the herbal extract could offer a safe and effective approach for skin regeneration and tissue repair.

The mixture exhibited anti-inflammatory effects, as evidenced by the reduction in *COX2* expression, indicating its potential for mitigating UV-induced skin inflammation. This effect may be comparable to dexamethasone, a known anti-inflammatory agent [48]. Additionally, HRMC5 significantly increased *Filaggrin* expression (*p* < 0.001), suggesting a role in enhancing skin barrier function. However, the lack of significant changes in *Claudin 1* expression implies that HRMC5 primarily influences barrier integrity through *Filaggrin* rather than uniformly affecting all barrier components. Overall, these findings suggest that HRMC5 could be beneficial in treating conditions characterized by inflammation and compromised skin barriers, warranting further investigation into its mechanisms and potential applications in dermatology.

Immunofluorescence staining and analysis showed that the mixture significantly upregulated four key skin barrier proteins: Involucrin, Filaggrin, Claudin 1, and Collagen Type 1. The effects were comparable to or exceeded those of the positive control (1% Glyceryl glucoside), a known enhancer of the skin barrier function [49]. Involucrin and Filaggrin are essential for skin hydration and preventing water loss [50], while Claudin 1 regulates the barrier integrity and permeability [51]. Collagen Type 1 provides structural support and skin elasticity [52]. These findings suggest that the mixture strengthens the skin barrier, improves hydration, and may contribute to enhanced skin elasticity and anti-aging effects, highlighting its potential in treating skin barrier dysfunction.

Our study on HRMC5-containing cream provides valuable insights into its efficacy in alleviating menopausal symptoms, particularly facial flushing, in postmenopausal women. We observed significant improvements in the skin parameters and menopausal symptoms over a 4-week period, suggesting that HRMC5 cream could be a promising topical treatment for managing these symptoms. We involved 20 participants, all in their 50s, with the majority experiencing severe menopause. This demographic is particularly relevant as menopausal symptoms, including facial flushing, are prevalent and often severe in this age group. The diverse skin characteristics of the participants, with a majority having dry skin and varying levels of facial moisture and oil, provided a comprehensive baseline for evaluating the cream’s effectiveness. We also considered lifestyle factors, such as daily UV exposure and skincare habits, to ensure that the observed effects were attributable to the cream rather than the external variables.

Our application of HRMC5 cream led to marked improvements in several key areas. We observed a progressive decrease in the skin redness and hemoglobin concentration on the cheeks, with significant reductions noted after 4 weeks. This suggests that the cream effectively targets the vascular components contributing to facial flushing. We also noted an increase in the skin moisture content, which is crucial for postmenopausal women who often experience dry skin due to hormonal changes. The significant rise in moisture levels indicates that HRMC5 cream may help restore the skin hydration balance. Our VAS evaluations showed substantial reductions in both facial flushing severity and the associated sweating. These findings highlight the potential of HRMC5 cream as a non-invasive treatment option for managing the vasomotor symptoms commonly experienced during menopause.

Our results align with other studies on plant-based treatments for menopausal symptoms [53]. For instance, the studies on phytoestrogens have shown reductions in hot flush frequency without serious side effects [53]. Moreover, a previous study examined the effects of genistein on hot flushes, which could be compared to the effects of HRMC5 cream on facial flushing and sweating [54]. Wild yam hormonal salves have also shown promise in alleviating menopausal symptoms such as hot flashes and joint discomfort [55]. However, unlike phytoestrogens which are ingested, HRMC5 cream offers a topical application method, potentially reducing the systemic side effects while providing localized relief.

We have demonstrated that HRMC5-containing cream is effective in reducing the facial flushing and improving the skin hydration among postmenopausal women. These findings suggest that HRMC5 cream could be a viable alternative or complement to the existing treatments for menopausal symptoms. Future research should explore the long-term efficacy and safety across larger populations to confirm these results and potentially expand its use to other menopausal symptoms. Additionally, understanding the mechanism by which HRMC5 exerts its effects could further enhance its application in the dermatological treatments for menopausal women.

## 5. Conclusions

Our comprehensive study of HRMC5 has yielded promising results across multiple aspects of its composition and therapeutic potential. The HPLC analysis identified 14 bioactive compounds, including flavonoids, phenolic acids, anthraquinones, and alkaloids, each contributing to the mixture’s therapeutic properties. The optimal concentration for cell survival and UV protection was determined to be 0.625 g/L. The herbal mixture demonstrated significant wound-healing properties comparable to EGF, exhibited strong anti-inflammatory effects by reducing *COX2* expression, and upregulated the key skin barrier proteins. In clinical application, the HRMC5-containing cream showed significant efficacy in alleviating menopausal symptoms, particularly facial flushing, in postmenopausal women over a 4-week period. We observed marked improvements in the skin redness, hemoglobin concentration, and skin moisture content, and substantial reductions in the facial flushing severity and associated sweating. These findings align with other studies on plant-based treatments for menopausal symptoms while offering the advantage of topical application. The results suggest that HRMC5 cream could be a promising non-invasive treatment option for managing the vasomotor symptoms in menopausal women. Future research should focus on the long-term efficacy, safety in larger populations, and elucidating the precise mechanisms of action.

## Figures and Tables

**Table 1:** Major compounds identified by HPLC analysis in HRMC5. The identified peaks and corresponding compounds from the HPLC analysis of HRMC5 are summarized. HRMC5 contains equal proportions of *Cimicifuga racemosa*, *Paeonia lactiflora*, *Phellodendron amurense*, *Rheum rhaponticum*, and *Scutellaria baicalensis* each individually extracted using 70% ethanol.

Peak Number	Retention Time (min)	Compound	Herb Source	Notable Properties
1	34.2	Paeoniflorin	*Paeonia lactiflora*	Anti-inflammatory
2	36.8	Polydatin	*Rheum rhaponticum*	Antioxidant, anti-inflammatory
3	42.3	Ferulic acid	*Cimicifuga racemosa*	Antioxidant, anti-aging
4	44.2	Isoferulic acid	*Cimicifuga racemosa*	Antioxidant
5	45.5	Rhein-glc	*Rheum rhaponticum*	Anti-inflammatory, antibacterial
6	47.9	Resveratrol-4′-(6″-galloyl glc)	*Rheum rhaponticum*	Antioxidant, skin protecting
7	53.5	Baicalin	*Scutellaria baicalensis*	Antioxidant, anti-inflammatory
8	56.7	Cimicifugic acid F	*Cimicifuga racemosa*	Anti-inflammatory
9	57.5	Berberine	*Phellodendron amurense*	Antibacterial, anti-inflammatory
10	58.5	Wogonoside	*Scutellaria baicalensis*	Antioxidant, skin soothing
11	64.5	Baicalein	*Scutellaria baicalensis*	Anti-inflammatory, antioxidant
12	71.2	Rhein	*Rheum rhaponticum*	Antimicrobial, anti-inflammatory
13	73.0	Wogonin	*Scutellaria baicalensis*	Anti-inflammatory, calming
14	86.4	Emodin	*Rheum rhaponticum*	Antibacterial, anti-inflammatory

**Table 2:** Skin characteristics of the research participants (*n* = 20).

Item	Category	Frequency	Percentage (%)
Age	20 s	0	0
	30 s	0	0
	40 s	0	0
	50 s	20	100
Skin type	Dry	11	55
	Normal	7	35
	Oily	0	0
	Combination	2	10
	Problematic	0	0
Facial moisture	Moist	0	0
	Normal	13	65
	Lacking	6	30
	Very lacking	1	5
Facial oil	Very Oily	0	0
	Normal	16	80
	Lacking	4	20
Body dryness	Moist	0	0
	Normal	11	55
	Dry	9	45
	Very dry	0	0
Shower frequency	1 or less	1	5
(per week)	2–3 times	1	5
	4–6 times	6	30
	Once daily	12	60
	2 or more times daily	0	0
Body product use	Do not use	4	20
	Occasionally use	15	75
	Always use	1	5
UV exposure	Less than 1 h	4	20
(per day)	1–3 h	15	75
	More than 3 h	1	5
Sleep duration	Less than 5 h	1	5
(per day)	5–8 h	18	90
	More than 8 h	1	5
Smoking status	Do not smoke	20	100
(per day)	Less than 10 cigarettes	0	0
	10 or more cigarettes	0	0
	More than a pack	0	0
Irritation sensitivity	Yes	0	0
	No	20	100
Stinging/itching	Yes	0	0
sensitivity	No	20	100
Adverse reaction	Yes	0	0
Experience	No	20	100
Skin changes	Yes	0	0
During menstruation	No	11	55
	Not applicable	9	45
Menstrual cycle	One week before period	1	5
	During period	0	0
	Within one week after period	2	10
	Other	8	40
	Not applicable	9	45

## Data Availability

Data are contained within the article.
